# The effect of acute sleep extension vs active recovery on post exercise recovery kinetics in rugby union players

**DOI:** 10.1371/journal.pone.0273026

**Published:** 2022-08-18

**Authors:** Cedric Leduc, Dan Weaving, Cameron Owen, Carlos Ramirez-Lopez, Sarah Chantler, Anis Aloulou, Jason Tee, Ben Jones

**Affiliations:** 1 Carnegie Applied Rugby Research (CARR) Centre, Carnegie School of Sport, Leeds Beckett University, Leeds, United Kingdom; 2 Leeds Rhinos Rugby League Club, Leeds, United Kingdom; 3 England Performance Unit, The Rugby Football League, Leeds, United Kingdom; 4 French National Institute of Sport (INSEP), Laboratory of Sport, Expertise and Performance (EA 7370), Paris, France; 5 Department of Physiology, Faculty of Health Sciences, University of Pretoria, Pretoria, South Africa; 6 School of Science and Technology, University of New England, Armidale, NSW, Australia; 7 Division of Exercise Science and Sports Medicine, Department of Human Biology, Faculty of Health Sciences, the University of Cape Town and the Sports Science Institute of South Africa, Cape Town, South Africa; Sheffield Hallam University, UNITED KINGDOM

## Abstract

**Background:**

Elite rugby players experience poor sleep quality and quantity. This lack of sleep could compromise post-exercise recovery. Therefore, it appears central to encourage sleep in order to improve recovery kinetics. However, the effectiveness of an acute ergogenic strategy such as sleep extension on recovery has yet to be investigated among athletes.

**Aim:**

To compare the effects of a single night of sleep extension to an active recovery session (CON) on post-exercise recovery kinetics.

**Methods:**

In a randomised cross-over design, 10 male rugby union players participated in two evening training sessions (19:30) involving collision activity, 7-days apart. After each session, participants either extended their sleep to 10 hours or attended an early morning recovery session (07:30). Prior to (PRE), immediately after (POST 0 hour [h]), 14h (POST 14) and 36h (POST 36) post training, neuromuscular, perceptual and cognitive measures of fatigue were assessed. Objective sleep parameters were monitored two days before the training session and over the two-day recovery period.

**Results:**

The training session induced substantial decreases in countermovement jump mean power and wellness across all time points, while heart rate recovery decreased at POST 0 in both conditions. Sleep extension resulted in greater total sleep time (effect size [90% confidence interval]: 5.35 [4.56 to 6.14]) but greater sleep fragmentation than CON (2.85 [2.00 to 3.70]). Between group differences highlight a faster recovery of cognitive performance following sleep extension (-1.53 [-2.33 to -0.74]) at POST 14, while autonomic function (-1.00 [-1.85 to -0.16]) and upper-body neuromuscular function (-0.78 [-1.65 to 0.08]) were better in CON. However, no difference in recovery status between groups was observed at POST 36.

**Conclusion:**

The main finding of this study suggests that sleep extension could affect cognitive function positively but did not improve neuromuscular function the day after a late exercise bout.

## Introduction

A growing body of evidence highlights that elite rugby players experience poor sleep quality and quantity following training and matches [1, 2]. Poor sleep patterns are likely explained by the different factors that occur before (*e*.*g*. caffeine consumption or supplementation), during (*e*.*g*. light exposure), and after (*e*.*g*. match outcomes, travel) a match or training session [3]. Each of these situations provides several stressors that induce specific psycho-physiological disturbances (*e*.*g*. muscle damage, change in core temperature) that can result in the reduction of sleep quality and quantity [4].

Fatigue is multidimensional and has been associated with a broad range of mechanisms and outcomes [4]. Due to this, a range of recovery mechanisms (e.g. glycogen resynthesis, muscle repair or mental relaxation) are involved in the restoration of homeostasis [5]. Many of these recovery mechanisms might be mediated by a lack of sleep [3]. Indeed, it appears that sleep loss compromises muscle recovery through creating greater catabolic states as well as negating energy store restoration [6, 7]. Therefore, due to the potential negative effects of sleep restriction after a match or training, it seems important to promote sleep to improve recovery kinetics. However, despite the widely held assumption that sleep aids the acute recovery process, the effectiveness of acute sleep extension on recovery is yet to be investigated among athletes [8].

Sleep extension (i.e. from 3 days to 11 weeks) [9, 10] has been described as the most efficient strategy to enhance sleep among athletes [11]. Yet, little is known regarding the usefulness of acute sleep extension on recovery kinetics. Despite the absence of clear evidence for its efficacy, acute sleep extension is prescribed in professional sport settings especially following late training sessions or matches [12]. An equally popular strategy to promote recovery following match play is the scheduling of morning recovery sessions [13]. Morning recovery sessions typically involve light aerobic activity, stretching and mobilisation [6]. However these recovery sessions might actually compound the sleep loss already encountered by players rather than improving their recovery due to early scheduling [14].

Therefore, the aim of this study was to assess the effect of one night of sleep extension on the physical, perceptual and cognitive recovery kinetics following late-night exercise compared to a traditional active recovery session prescribed early in the morning.

## Materials and methods

### Subjects

Twelve male rugby union players competing in university rugby competition were initially recruited. Two players were removed from the study due to the lack of compliance in the sleep extension protocol. 10 players were finally included (age: 21.0 ± 1.3 years; height: 179.9 ± 5.4 cm; body mass: 89.2 ± 15.4 kg). Players selected for this study were all regularly involved in late training sessions or matches. Participants provided informed consent prior to starting the study. Ethics approval was granted by the Leeds Beckett University ethics board and the recommendations of the Declaration of Helsinki were respected. Each participant provided written consent before the beginning of the study.

### Design

**Fig 1:**
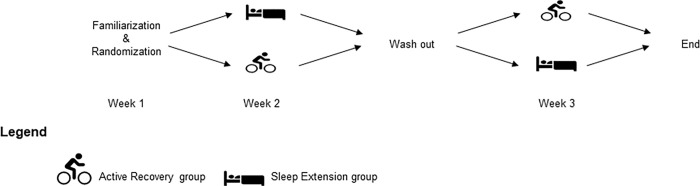
General study design.

A randomised single-blind cross-over design was used to determine the effects of sleep extension or active recovery sessions on changes in physical and psychological recovery measures following a late standardized training session (Fig 1). During week 1, the participants were familiarised with the testing procedure on two occasions, 48 hours (h) apart. Following this familiarisation period, participants were randomly assigned to either the sleep extension group (SE) or the active recovery (control; CON) group. The lead investigator was blinded from subject allocation in order to reduce risk of bias during data collection.

**Fig 2:**
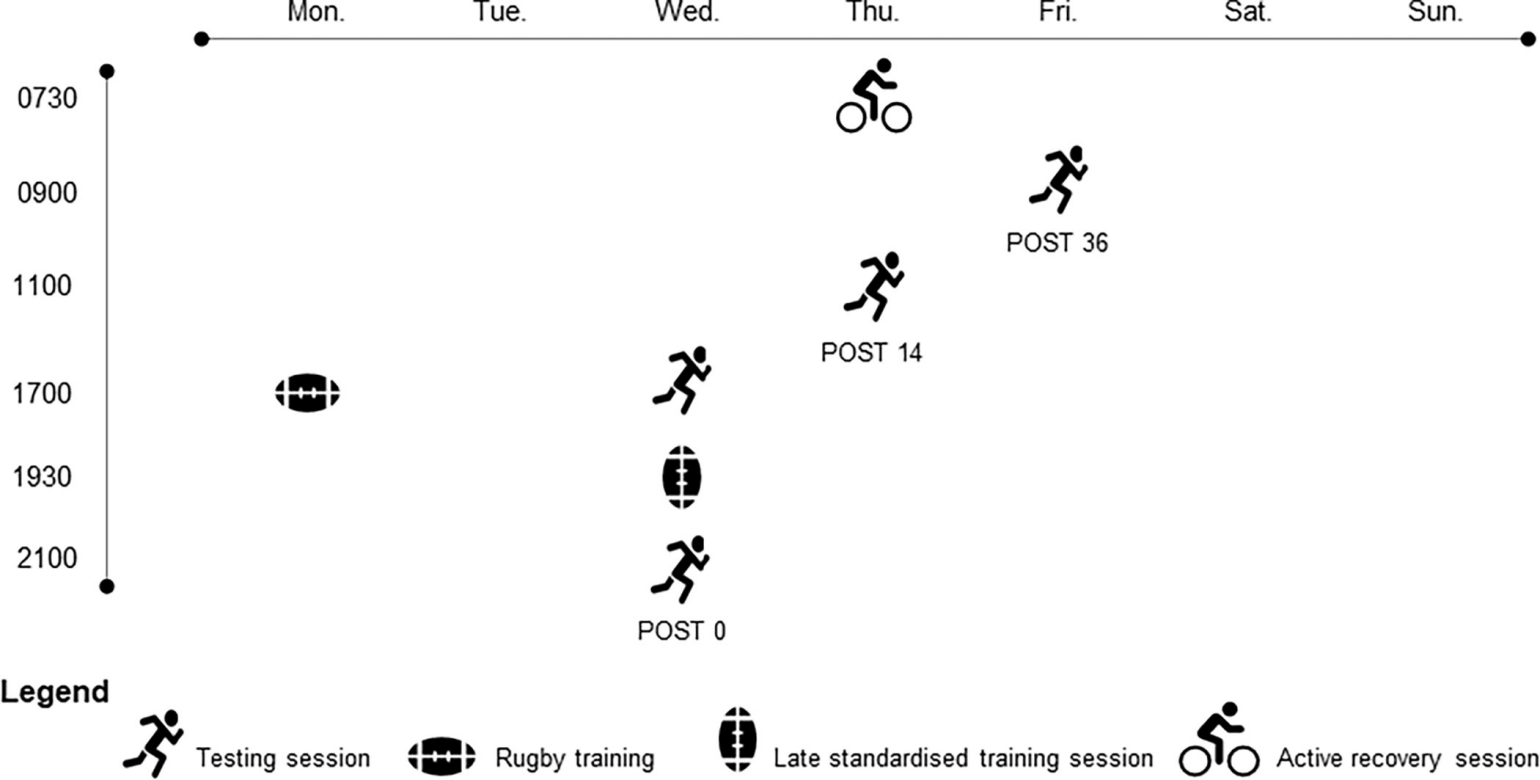
Weekly organisation of the study.

The weekly organisation of the study is displayed in Fig 2. On day one, participants performed a standardized skills session at 14:00. On day two, no training was conducted to maintain a similar physiological status over the two weeks. On day three, the late night standardized training session was performed on a natural grass surface at 19:30. Baseline testing was conducted before (17:00—PRE). Post testing was performed immediately after the session (21:00—POST 0). Following the late standardized training session the SE were advised to spend at least 10 hours in bed the night after the training session (night 1) based on the recommendations by Mah et al. [15]. To achieve this, the SE group was asked to wake up at 10:00 in order to ensure a sufficient sleep extension window. The CON group was asked to wake up at 06:00. On day four, participants who were assigned to the CON group performed the recovery session at 07:30. Both CON and SE groups performed the first post-test 14h after the training session (POST 14) at 11:00. Both groups had the same sleep schedule without any other particular instructions between day four and five (night 2). At 09:00 on day five, participants performed the final testing session corresponding to 36h post training session (POST 36). The same procedure was conducted on week 2 and 3 with 3 days of full recovery between each week. Between week 2 and 3 no additional training was performed by the players.

#### Standardized late training session

A standardised full contact training session was prescribed and delivered by experienced coaches. This standardised contact session has previously been used within a rugby union playing population and demonstrated a capability to induce a substantial fatigue response [16]. After a standardized warm up, each team played three 3 minute small-sided games (4 vs. 4 players; 15 x 20 m pitches) with 90s of rest in between each game. Following this, players were then divided into two teams (8 vs. 8) and performed another sequence of three 3 min small-sided games with 90s recovery. The order of games and members of teams were kept the same for both sessions (week 2 and 3). Six additional players were used but not included in the study.

During each session, participants wore the same GPS unit (Optimeye S5, Catapult Innovations, Melbourne, Australia) and heart rate monitor (Polar T31 coded, Kempele, Finland) used during on field testing. Total distance covered (TD), high-speed distance (HSD >5 m^.^s^-1^) and sprint (> 7 m^.^s^-1^) in meters (m) and PlayerLoad^TM^ (Arbitrary Units [AU]) were used to quantify external training load. Average HR and session rating of perceived exertion (sRPE) using Borgs CR10 scale was used to quantify internal load. The sRPE was collected within 30 min after each session [17] and was then multiplied by training duration to provide sRPE-TL [17]. Finally, collision frequency was quantified through notational analysis by an experienced video analyst.

#### Recovery and sleep extension intervention

The sleep extension protocol involved extending time in bed up to 10 hours of sleep as recommended by previous studies [15]. To achieve this, verbal and written instructions for sleep scheduling were provided to the participants (i.e. time to go to bed and fall asleep, time to wake up time). No recovery session was scheduled for this group, who were only required to return for testing at 11:00 the following morning, thus providing the opportunity for extended sleep. The active recovery session started at 07:30. The session was as follows: 1.) 15 min continuous bout on the bike at a controlled intensity (50% maximum heart rate, determined by the equation of Tanaka et al. [18]) and 2.) a combination of standardised stretching and mobility exercises for both upper and lower body was performed for 15 min. This session was led by an experienced strength and conditioning coach and is typical of the recovery protocols rugby teams might engage in. To avoid confounding factors, nutrition was also controlled during the study period. The evening meal was standardized for both groups after the training session (22:30) and was provided by the research team to ensure similar nutritional intake for each participant. For both groups, breakfast on day four and five were standardized and caffeine was prohibited in order to control the potential effect of nutrition on recovery status. For lunch and dinner, players were asked to take a picture of their meals and replicate them over the two-week study period [19].

#### Sleep monitoring

Sleep was recorded with a wristwatch actigraphy from day one to day five. Participants were allocated an Actiwatch MotionWatch 8 (Cambridge Neurotechnology Ltd., Cambridge, UK) which was worn on the non-dominant wrist. A medium threshold (>40 activity counts scored as wake) was used for sleep analysis, as recommended by Fuller et al. [20] for team-sport athletes. The sleep variables were calculated as described by the software MotionWare 1.1.25 (Cambridge Neurotechnology Ltd., Cambridge, UK) and are presented in Table 1. Data gathered in the 2 nights preceding the standardized training session were pooled in order to create baseline value.

**Table 1:** Session training load.

	sRPE-TL (A.U)	Average HR (BPM)	Total distance (m)	Low speed distance (m)	High speed distance (m)	Sprint distance (m)	PlayerLoad^TM^ (A.U)	Collision activity (n)
Week 1	350 ± 82	168 ± 9	3120 ± 203	2512 ± 118	540 ± 103	144 ± 40	371 ± 41	22 ± 9
Week 2	311 ± 49	166 ± 8	3166 ± 331	2562 ± 215	531 ± 118	132 ± 34	390 ± 66	23.7 ± 6.5
CV(%)	27.1 (19.1 to 48.4)	2.1 (1.5 to 3.5)	5.6 (4.0 to 9.3)	5.1 (3.7 to 8.5)	11.5 (8.3 to 19.6)	25.3 (17.9 to 44.9)	7.5 (5.4 to 12.7)	15.7 (11.3 to 27.2)

The training load data are presented as mean ± SD. The changes between Week 1 and 2 are presented as typical error expressed as coefficient of variation (CV%) and 90% confidence intervals. sRPE-TL stands for session rating of perceived exertion–training load. A.U stands for arbitrary units. HR stands for heart rate. BPM stands for beats per minute.

### Recovery testing procedures

#### Perceptual measures

A 5-item questionnaire from McLean et al. [21] to rate sleep quality, fatigue, muscle soreness, stress and mood on a 5-point Likert scale was used. Overall wellness was assessed by summing the 5 scores. Reliability of the questionnaire has been assessed in a previous study and showed a coefficient of variation of 7.1% (5.8% to 9.1%) [22].

#### Cognitive function

A Stroop test was used to assess cognitive function as it involves brain areas such as the dorsolateral prefrontal cortex and the anterior cingulate cortex [23] which have been shown to be influenced by sleep loss [24]. The test was performed on EncephalApp (EncephalApp, HindSoft Technology, India) and performed on an electronic tablet (Ipad, Apple, USA). Recent literature review reporting the test-retest reliability of a computerized Stroop task highlighted good level of reliability (ICC>0.90) [25]. Participants performed one set comprising 10 repetitions. Participants were required to answer appropriately to a series of colour words under conditions where word meanings and ink colours are incongruent (e.g. the word green displayed in red ink) or congruent (e.g. the word red printed in red ink). Before each session, two practice tests were performed to avoid any learning effects. For each session, participants completed the testing in an isolated room to avoid any disturbances. Each participant was asked to be seated and perform the test with the same hand throughout the study. The time to complete the task was used for analysis.

#### Autonomic function

Players completed 5 min of running 20 m shuttles at a submaximal running speed fixed at 9 km∙h^-1^, followed immediately by 2 min of passive recovery during which heart rate recovery (HRR) was determined as described by Buchheit et al. [26]. Post-exercise HR reflects general hemodynamic adjustments and is related to readiness to perform [26]. Recent investigation showed a coefficient of variation of 3.4% among similar rugby league players [27].

#### Locomotor efficiency

Following the sub-maximal running test, participants performed four paced, high speed runs to allow for the calculation of running load index (RLI) as a measure of locomotor efficiency as per previous methods [28]. Each run was 60 m and players were paced to complete the run in 12 seconds (mean velocity ≈ 18 km·h^-1^). This method has demonstrated a large relationship with leg stiffness (r = 0.62) as well as a small typical error (= 0.54) [28].

#### Neuromuscular function

Countermovement jump (CMJ) and plyometric push-up (PP) were performed on a force plate (PASPORT force plate, PS-2141, PASCO Scientific, California, USA). CMJ mean power and PP flight-time were used for analysis as they have been deemed as reliable markers of fatigue in rugby union [22].

### Statistical analysis

All data were log transformed to reduce bias as a result of nonuniformity error. Data were analysed using linear mixed models within R Studio (Version 1.1.442, R Foundation for Statistical Computing). Time course of recovery within group were assessed by running two separate models and considering period of assessment (POST 0, POST 14, POST 36) as the fixed effect and pre training session (PRE) testing battery as reference for comparison. Regarding the effect of conditions (SE vs. CON), between groups comparisons were performed using the condition as fixed effect. In all models, participants were considered as a random factor. Differences were assessed with the least square means test and further with standardized effect sizes (ES) and 90% confidence interval (CI) derived from mixed model analysis. Further comparisons were then performed with a magnitude-based decision framework (MBD) [29]. The ES magnitude was classified as trivial (<0.2), small (0.2–0.6), moderate (>0.6–1.2), large (>1.2–2.0) and very large (>2.0–4.0) (Hopkins, 2009). The effects were considered practically meaningful if the likelihood of a true effect was > 75%.

## Results

### Standardized late training session

The session training load are presented in Table 2.

**Table 2:** Definitions of each sleep variable derived from the wrist watch actigraphy.

Sleep variables (units)	Definition
Bed time (hh:mm)	Estimated clock time at which the player attempts to sleep (press the button marker)
Fall asleep time (hh:mm)	Estimated clock time at which the player fell asleep
Wake time (hh:mm)	Estimated clock time at which the player woke up
Get up time (hh:mm)	Estimated clock time at which the player stops sleeping (press the button marker)
Time in Bed (min)	Time between bed time and get up time
Sleep onset latency (min)	Time between bed time and sleep onset
Total sleep time (min)	Time spent asleep determined from sleep onset to wake up time, minus any wake time
Wake after sleep onset (WASO) (%)	The total time spent in wake according to the epoch-by-epoch wake/sleep categorization expressed as % of total sleep time
Sleep efficiency (%)	Total sleep time divided by the time in bed
Fragmentation index (%)	Sum of the mobile time (%) and the immobile bouts ≤1 min

### Sleep parameters

**Fig 3:**
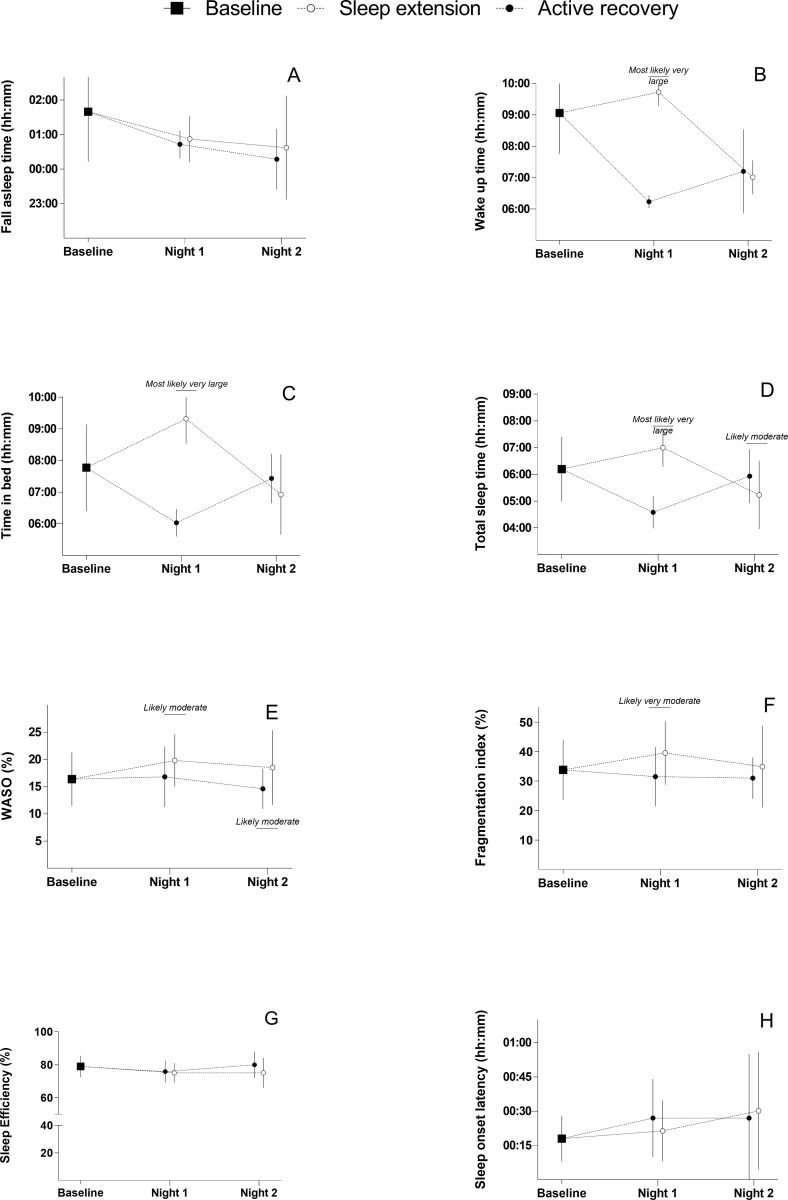
Between group comparison for sleep parameters. White and black dots represent the sleep extension and active recovery group respectively. Error bars represent standard deviations. WASO: wake after sleep onset. Figure A: Fall asleep time. Figure B: Wake up time. Figure C: Time in bed. Figure D: Total sleep time. Figure E: WASO. Figure F: Fragmentation index. Figure G: Sleep Efficiency. Figure H: Sleep onset latency.

Table 3 shows the baseline values for sleep and associated differences for each condition. Fig 3 shows between group differences in sleep parameters at each timepoint. In the night following the training session, SE showed a *most likely* later wake up time (ES [90% CI] = 10.20 [9.46 to 10.94]) than CON (p<0.01). This also led to a *most likely* longer time in bed (p<0.01; 7.24 [6.44 to 8.05]), total sleep time (p<0.01; 5.35 [4.56 to 6.14]) and likely higher WASO (p>0.05; 1.03 [0.18 to 1.89]) for SE. Although, a *likely* worst sleep efficiency was observed in SE (p>0.05; 0.99 [0.14 to 1.84]). Differences between SE and CON for fall asleep time (p>0.05; 0.44 [-0.41 to 1.28]) and sleep latency (p>0.05; -0.39 [-1.24 to 0.45]) were deemed *unclear*. For the second night following the training session, a *likely* shorter total sleep time was observed in SE compared to CON (p>0.05; -0.73 [-1.59 to 0.12]). Other results were deemed *unclear*.

**Table 3:** Sleep parameters during baseline, night 1 and 2.

	Baseline	Condition	Night 1	Night 2	Baseline to Night 1	Baseline to Night 2
					d (±90% CI)	d (±90% CI)
Fall asleep time (hh:mm)	01:39 ± 01:27	CON	00:43 ± 00:23	00:16 ± 00:52	p>0.05	P<0.05
					0.62 (-0.02 to 1.27)*	0.91 (0.27 to 1.55)**
		SE	00:52 ± 00:39	00:37 ± 01:30	p>0.05	p>0.05
					-0.54 (-1.16 to 0.08)*	-0.62 (-1.23 to 0.00)*
Wake up time (hh:mm)	09:03 ± 01:17	CON	06:13 ± 00:11	07:12 ± 00:10	P<0.01	P<0.01
					2.84 (2.20 to 3.48)***	1.98 (1.34 to 2.63)***
		SE	09:43 ± 00:26	07:00 ± 00:32	p>0.05	P<0.01
					0.49 (-0.13 to 1.11)*	-1.90 (-2.52 to -1.29)***
Time in bed (hh:mm)	07:46 ± 01:22	CON	06:01 ± 00:26	07:25 ± 00:46	P<0.01	p>0.05
					1.35 (0.71 to 1.99)***	0.40 (-0.25 to 1.04)
		SE	09:19 ± 00:46	06:55 ± 01:16	P<0.01	p>0.05
					0.99 (0.38 to 1.61)**	-0.69 (-1.31 to -0.07)*
Total sleep time (hh:mm)	06:11 ± 01:12	CON	04:35 ± 00:36	05:55 ± 00:59	P<0.01	p>0.05
					1.32 (0.68 to 1.96)***	0.31 (-0.34 to 0.95)
		SE	07:00 ± 0:43	05:13 ± 01:16	p>0.05	P<0.05
					0.56 (-0.06 to 1.17)*	-0.76 (-1.38 to -0.15)*
WASO (%)	16.4 ± 4.9	CON	16.8 ± 5.6	14.6 ± 3.7	p>0.05	p>0.05
					0.05 (-0.60 to 0.69)	-0.59 (-1.23 to 0.06)**
		SE	19.8 ± 4.8	18.5 ± 6.9	P<0.01	p>0.05
					1.74 (1.12 to 2.36)***	0.69 (0.07 to 1.31)*
Fragmentation index (%)	33.8 ± 10.2	CON	31.5 ± 10.0	31.3 ± 7.2	p>0.05	p>0.05
					0.61 (-0.03 to 1.26)*	0.63 (-0.02 to 1.27)*
		SE	39.6 ± 10.7	34.9 ± 13.8	p>0.05	p>0.05
					0.67 (0.05 to 1.30)*	-0.04 (-0.66 to 0.58)
Sleep efficiency (%)	79.0 ± 6.4	CON	75.9 ± 6.7	79.6 ± 7.6	p>0.05	p>0.05
					0.36 (-0.28 to 1.00)	-0.15 (-0.79 to 0.49)
		SE	75.1 ± 5.9	75.1 ± 9.0	p>0.05	p>0.05
					-0.65 (-1.27 to -0.02)*	-0.50 (-1.12 to 0.12)*
Sleep latency (hh:mm)	00:18 ± 00:10	CON	00:27 ± 00:16	00:27 ± 00:28	p>0.05	p>0.05
					-0.42 (-1.06 to 0.22)	-0.38 (-1.02 to 0.26)
		SE	00:21 ± 00:13	00:30 ± 00:25	p>0.05	p>0.05
					0.12 (-0.49 to 0.74)	0.51 (-0.10 to 1.12)*

Data are presented as mean ± SD. Within group differences with Baseline are presented as effect size and 90% confidence intervals.

*: likely

**: very likely

***: most likely change/difference between group. SE and CON stand for Sleep Extension and CONTROL respectively. WASO stands for wake after sleep onset.

### Time course recovery of perceptual, cognitive and physical parameters

The time-course of the various recovery parameters for each condition are displayed in Tables 4 and 5. From POST 0 to POST 14, perceptual ratings of fatigue and soreness were *most likely* to *likely* greater than PRE for both conditions. Consequently, this resulted in *very likely* to *likely* lower total score over the same period. *Likely* better mood and *likely* worse sleep quality were observed at POST 36 than PRE for CON and SE respectively. Other results were deemed *unclear*. At POST 14, the time to complete the Stroop task was *likely* shorter in the SE condition compared with PRE. At POST 0, *most likely to very likely* lower HRR values were observed in both conditions. At POST 14, *likely* lower HRR was found in SE condition. At POST 36, *likely* higher HRR were observed in SE. *Unclear* differences were found with RLI at POST 0 compared with PRE. At POST 14, *most likely* to *very likely* higher RLI were found in both conditions, at POST 36 only the SE condition demonstrated a *most likely* higher RLI. *Most likely* to *likely* lower mean power produced during CMJ were observed from POST 0 to POST 36 in both conditions. Only a *likely* shorter flight time during PP was observed in the SE condition. Other results for this variable were deemed *unclear*.

**Table 4:** Wellness scores change as percentage of PRE.

	Condition	Baseline	POST 0	POST 14	POST 36	Baseline to POST 0	Baseline to POST 14	Baseline to POST 36
						d (±90% CI)	d (±90% CI)	d (±90% CI)
Wellness–Fatigue (A.U)	CON	3.1 ± 0.8	-31.5 ± 19.4%	-19.4 ± 31.7%	-15.7 ± 36.0%	P<0.01	P<0.05	P>0.05
						-2.01 (-2.85 to 1.17)***	-1.19 (-2.01 to -0.36)**	-0.88 (-1.71 to 0.06)*
	SE	3.0 ± 0.8	-25.0 ± 32.6%	-11.7 ± 15.3%	-5.6 ± 11.0%	P<0.05	P<0.05	P>0.05
						-1.28 (-2.08 to -0.48)**	-1.10 (-1.92 to -0.28)**	-0.64 (-1.52 to 0.23)
Wellness–Sleep (A.U)	CON	3.3 ± 0.9	-2.8 ± 8.3%	-11.1 ± 15.3%	10.2% ± 0.9	P<0.05	P<0.05	P<0.05
						-0.47 (-1.35 to 0.41)	-0.54 (-1.42 to 0.33)	0.38 (-0.50 to 1.25)
	SE	3.7 ± 1.1	11.3 ± 33.7%	-4.8 ± 39.4%	-10.7 ± 23.8%	P<0.05	P<0.05	P<0.05
						0.36 (-0.46 to 1.18)	-0.34 (-1.16 to 0.48)	-0.81 (-1.67 to 0.04)*
Wellness–Soreness (A.U)	CON	3.1 ± 1.1	-32.6 ± 25.4%	-21.5 ± 36.3%	-10.0 ± 43.9%	P<0.01	P<0.05	P>0.05
						-1.49 (-2.35to -0.63)**	-1.01 (-1.83 to -0.19)*	-0.59 (-1.47 to 0.29)
	SE	3.4 ± 0.7	-50.0 ± 16.2%	-23.3 ± 20.3%	-7.4% ± 26.2%	P<0.01	P<0.01	P>0.05
						-3.56 (-4.38 to -2.74)***	-1.79 (-2.61 to -0.97)***	-0.63 (-1.49 to 0.23)
Wellness–Stress (A.U)	CON	3.7 ± 0.9	2.0 ± 29.7%	-5.0 ± 29.1%	-1.3 ± 40.7%	P>0.05	P>0.05	P>0.05
						-0.15 (-1.04 to 0.74)	-0.54 (-1.42 to 0.33)	-0.32 (-1.20 to 0.55)
	SE	3.7 ± 1.3	22.5 ± 134.7%	-0.2% ± 41.4%	3.9% ± 37.6%	P>0.05	P>0.05	P>0.05
						-0.34 (-1.12 to 0.43)	-0.59 (-1.41 to 0.23)	-0.49 (-1.36 to 0.38)
Wellness–Mood (A.U)	CON	3.4 ± 0.7	12.0 ± 36.1%	7.4 ± 26.2%	9.3 ± 18.8%	P>0.05	P>0.05	P>0.05
						0.38 (-0.51 to 1.26)	0.20 (-0.69 to 1.09)	0.71 (-0.16 to 1.59)*
	SE	3.7 ± 1.3	25.0 ± 132.8%	8.5 ± 49.4%	12.2% ± 38.7%	P>0.05	P>0.05	P>0.05
						-0.17 (-1.00 to 0.65)	-0.27 (-1.09 to 0.55)	-0.02 (-0.89 to 0.85)
Wellness–Total (A.U)	CON	16.7 ± 3.2	-11.1% ± 15.4%	-10.9 ± 19.5%	-3.9 ± 22.1%	P<0.05	P>0.05	P>0.05
						-1.08 (-1.90 to -0.26)**	-0.81 (-1.63 to 0.01)*	-0.39 (-1.21 to 0.44)
	SE	17.5 ± 3.5	-11.9 ± 33.5%	-10.2 ± 17.4%	-14.5 ± 33.2%	P<0.05	P>0.05	P>0.05
						-1.03 (-1.85 to -0.22)**	-0.90 (-1.72 to -0.08)*	-0.75 (-1.57 to 0.07)*

Data are presented as mean ± SD. Within group differences with PRE are presented as effect size and 90% confidence intervals.

*: likely

**: very likely

***: most likely change/difference between group. SE and CON stand for Sleep Extension and CONTROL respectively. A.U stands for arbitrary units.

**Table 5:** Cognitive and physical markers change as percentage of PRE.

	Condition	Baseline	POST 0	POST 14	POST 36	Baseline to POST 0	Baseline to POST 14	Baseline to POST 36
						d (90% CI)	d (90% CI)	d (90% CI)
Stroop test (s)	CON	10.00 ± 2.17	-1.7 ± 8.73%	4.9 ± 9.5%	-0.6 ± 7.9%	P>0.05	P>0.05	P>0.05
						-0.33 (-1.21 to 0.54)	0.66 (-0.22 to 1.54)	-0.24 (-1.12 to 0.64)
	SE	10.44 ± 1.70	-1.78 ± 11.3%	-9.0 ± 8.39%	-1.7 ± 11.4%	P>0.05	P<0.05	P>0.05
						-0.20 (-1.02 to 0.62)	-1.38 (-2.20 to -0.56)**	-0.24 (-1.11 to 0.63)
HR-recovery (BPM)	CON	23.10 ± 7.60	-21.3 ± 12.1%	28.5 ± 45.0%	21.3 ± 50.2%	P<0.01	P>0.05	P>0.05
						-2.33 (-3.20 to -1.45)***	0.56 (-0.31 to 1.44)	0.26 (-0.62 to 1.14)
	SE	23.30 ± 7.10	-21.9 ± 21.6%	-7.7 ± 16.5%	6.7± 11.9%	P<0.05	P>0.05	P>0.05
						-1.32 (-2.14 to -0.50)**	-0.70 (-1.52 to 0.12)*	0.77 (-0.11 to 1.65)*
RLI (A.U)	CON	50.80 ± 17.10	23.0 ± 44.9%	26.1 ± 27.6%	14.8 ± 34.0%	P>0.05	P<0.05	P>0.05
						0.73 (-0.22 to 1.68)	1.78 (0.75 to 2.80)**	0.60 (-0.35 to 1.55)
	SE	47.90 ± 12.50	5.6 ± 11.7%	20.5 ± 10.2%	16.7 ± 11.3%	P>0.05	P<0.01	P<0.01
						0.55 (-0.33 to 1.43)	2.91 (1.75 to 4.06)***	2.39 (1.51 to 3.27)***
Mean power CMJ (W)	CON	2575 ± 408	-5.8 ± 5.5%	-8.6 ± 8.6%	-8.7 ± 8.5%	P<0.01	P<0.01	P<0.01
						-1.51 (-2.39 to -0.63)**	-1.57 (-2.44 to -0.69)**	-1.47 (-2.34 to 0.59)**
	SE	2583 ± 455	-7.6% ± 10.3%	-7.8% ± 11.5%	-6.2 ± 4.3%	P<0.05	P>0.05	P<0.01
						-1.17 (-1.99 to -0.35)**	-0.97 (-1.78 to -0.15)*	-2.03 (-2.90 to 1.15)***
PP flight time (s)	CON	0.28 ± 0.04	-0.5 ± 10.0%	-1.2% ± 17.9%	-3.5 ± 20.4%	P>0.05	P>0.05	P>0.05
						-0.09 (-0.97 to 0.78)	-0.13 (-1.01 to 0.74)	-0.25 (-1.13 to 0.62)
	SE	0.27 ± 0.05	4.3 ± 10.4%	-8.5 ± 18.1%	1.7 ± 10.9%	P>0.05	P>0.05	P>0.05
						0.51 (-0.31 to 1.33)	-0.97 (-1.79 to -0.15)*	0.02 (-0.85 to 0.90)

Data are presented as mean ± SD. Within group differences with PRE are presented as effect size and 90% confidence intervals.

**: likely

***: very likely

****: most likely change/difference between group. SE and CON stand for Sleep Extension and CONTROL respectively. BPM stands for beats per minute. A.U stands for arbitrary units. CMJ stands for counter movement jump. PP stands for plyometric push-up.

### Effect of condition on perceptual, cognitive and physical parameters

**Fig 4:**
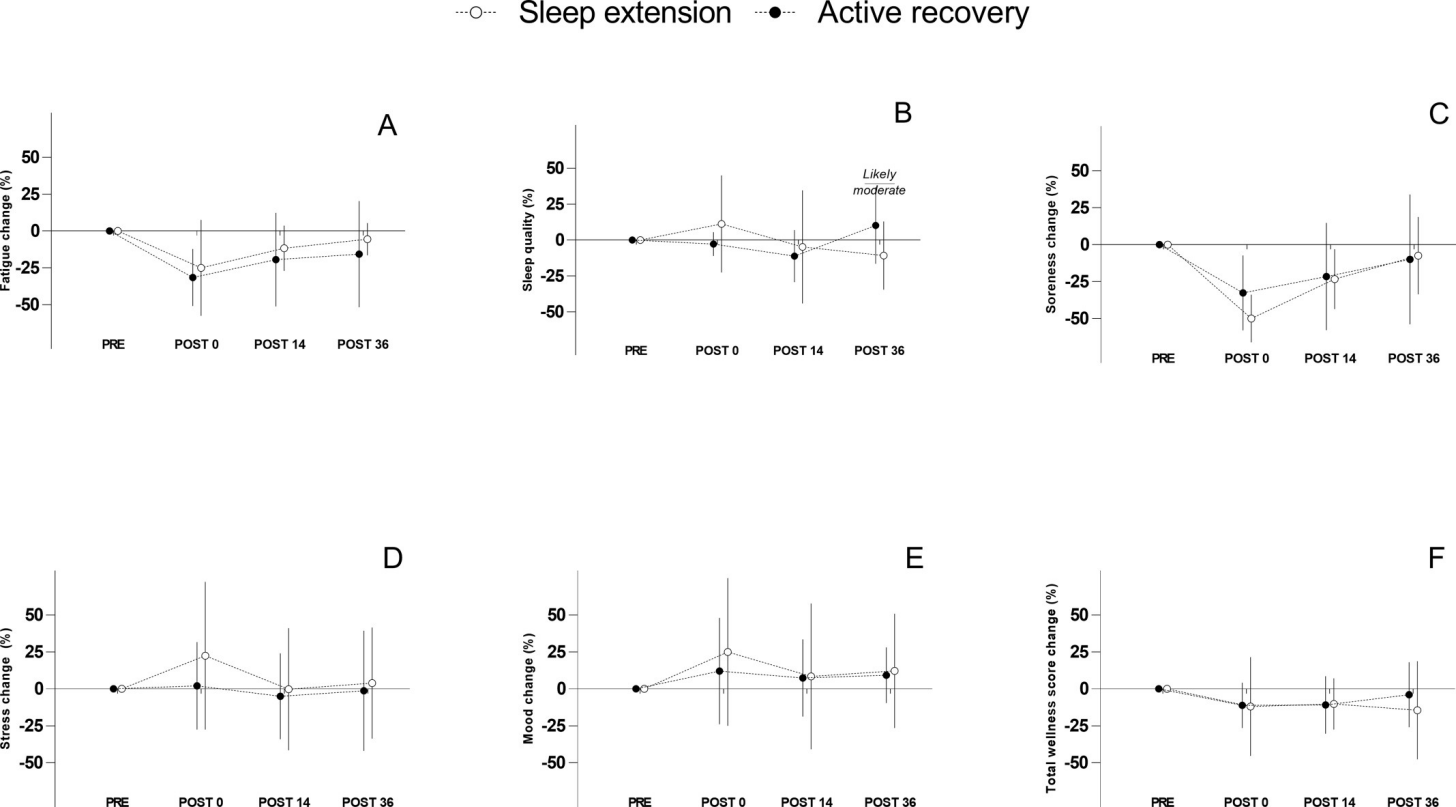
Between group comparison for wellness scores. White and black dots represent the sleep extension and active recovery group respectively. Error bars represent standard deviations. Figure A: Fatigue. Figure B: Sleep quality. Figure C: Soreness. Figure D: Stress. Figure E: Mood. Figure F: Total wellness score.

**Fig 5:**
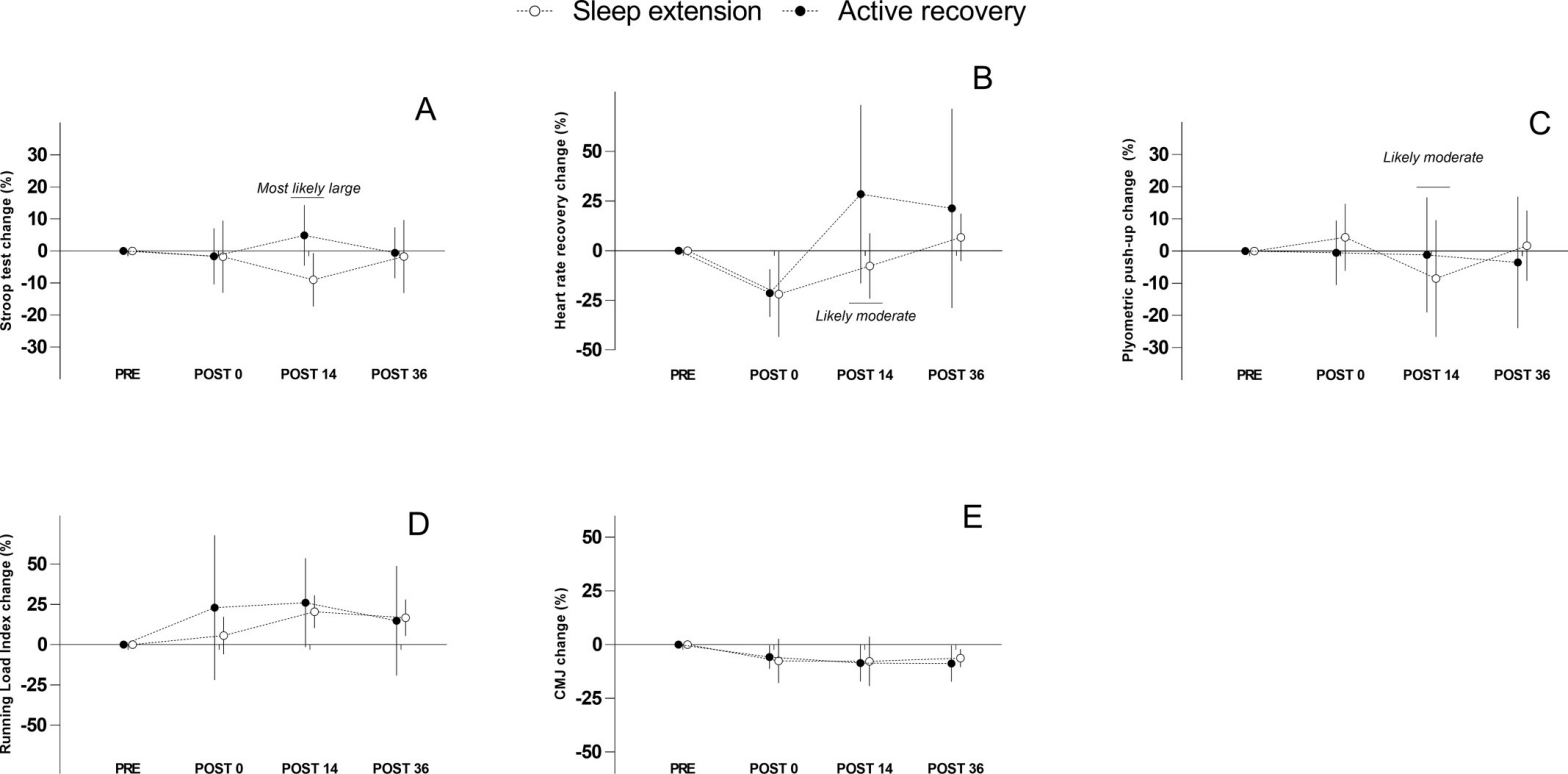
Between group comparison for physical and cognitive markers. White and black dots represent the sleep extension and active recovery group respectively. Error bars represent standard deviations. Figure A: Stroop test. Figure B: Heart rate recovery. Figure C: Plyometric push-up. Figure D: Running load index. Figure E: Counter movement Jump.

Differences between conditions are displayed in Figs 4 and 5. Regarding the wellness questionnaire, only a *likely* better sleep quality was observed at POST 36 in favour of the CON condition (p>0.05; -0.83 [-1.65 to -0.01]). Other results regarding the wellness questionnaire were deemed *unclear*. At POST 14, a *most likely* shorter time to complete the Stroop task was observed in the SE condition compared to CON (p<0.01; -1.53 [-2.33 to -0.74]). Conversely, HRR was *likely* better in the CON condition compare with SE (p<0.05; -1.00 [-1.85 to -0.16]). Similarly, *likely* longer flight time performed during PP was observed in the CON condition (p>0.05; -0.78 [-1.65 to 0.08]). No effect of the condition was observed for RLI and CMJ either at POST 14 or POST 36.

## Discussion

The aim of this study was to assess the effect of one night of sleep extension on recovery kinetics compared to an active recovery session prescribed early in the morning. The main finding of this study was that at 14h post training (POST 14), sleep extension resulted in improved cognitive performance, while active recovery resulted in improved upper body neuromuscular and autonomic function. However, at 36h post training (POST 36) no substantial differences between conditions were present. These findings suggest that the effects of acute recovery interventions are in themselves acute, and that recovery interventions will have varied effects on the diverse aspects of recovery during this acute period. Consequently, if athletes must perform again within short timeframes (e.g. multi days event, congested fixtures), practitioners can consider which recovery protocols that best align to the performance requirements. In cases where longer recovery is possible, it remains unknown if a sleep extension performed over several days would be more beneficial than active recovery.

### Standardized training session and acute responses

The results of the current study suggest that the standardised training session induced immediate (i.e. POST 0) and substantial changes in perceptual fatigue measures, neuromuscular function and HRR. Neither cognitive nor upper body neuromuscular parameters were affected by the training exposure at POST 0. The decreases in perceptual fatigue and lower body neuromuscular function were persistent at POST 14 and POST 36. Roe et al. [16] reported similar results regarding perceptual recovery despite different responses regarding the neuromuscular function. While Roe et al. [16] observed a change in upper body neuromuscular function, in the present study, only lower body neuromuscular function was affected. The reason for this difference remains hypothetical but could be related to the player level (academy vs. university). No between group differences were observed at POST 0 for any measure indicating that the training exposure caused similar fatigue effects in both conditions. Collectively, these results suggest that the standardized training session is a reliable protocol to induce substantial fatigue responses over two days and could be used for future research.

### The effects of sleep extension and active recovery on sleep parameters at night 1

During night 1, the SE intervention elicited a very large increase in the time spent in bed and consequently total sleep time in comparison to CON. However, while time in bed improved, total sleep time only demonstrated a small improvement compared to baseline (≈ 1 hour) reaching only the 7h of total sleep time which is line with recommendation for healthy individual [30] but potentially below what is recommended and needed for athletes [31, 32]. In a similar study [9], participants increased total sleep time only by 1h after a football match played in the evening (i.e. match finished at 22:30). These results suggest that it is difficult to acutely extend total sleep time by more than 1h in team sport athletes. This may be because the rapid change in sleep schedule conflicts with established circadian rhythms [33]. While in this study, participants slept in their home environment, in practical settings, elite athletes often slept in different environment could also limits the integration of sleep extension for this population. Furthermore, poorer sleep on Night 2 (i.e. shorter total sleep time, increased Fragmentation Index and WASO) for the SE group suggesting that sleep extension may have some detrimental effects on the subsequent night of sleep. Consequently, due to the potential difficulties to increase sleep acutely in the context of team sport, practitioners should consider keeping consistent sleep patterns and potentially add a post lunch nap during the day following a late exercise if a decrease in total sleep time is observed. Several studies have highlighted that when sleep restriction occurs, napping could be an efficient strategy to maintain total sleep time [1] but also performance [34, 35]. However, guidance regarding nap timing (e.g. between 13:00 to 16:00) and duration (e.g. <30min) need to be provided to the athletes [31]. Moreover, the increase in fragmentation index, as well WASO was observed, suggesting that sleep was more disturbed in SE and may lead to restless sleep. Those results suggest that sleep quality should be improved concurrently to the sleep extension protocol. The importance of improving sleep quality is further reinforced by recent findings showing that collision activity could deteriorate sleep architecture [36].

### The effects of sleep extension and active recovery on recovery at POST 14

The main results showed that SE affected cognitive function positively, although there was no improvement in recovery status of the lower body neuromuscular function at POST 14. Similarly, Fullagar et al. [9] found that an acute sleep hygiene strategy did not improve physical recovery after a football match. They hypothesised that neuromuscular function failed to improve because difference in terms of sleep quantity (i.e. total sleep time) was not sufficient (≈1h). However, in the current study the effective difference in total sleep time between groups was much larger (≈ 02h30), but still did not result in any improvement in lower body neuromuscular function. This suggests that recovery of neuromuscular function following training and collision exposure is not sensitive to acute changes in total sleep time.

The difference in cognitive function between groups in the current study is not surprising. It is well documented that sleep restriction has deleterious consequences for cognitive function [37]. The CON group’s total sleep time was low (≈ 04h30) which likely caused the large between group difference. Despite the absence of a substantial between group difference, HRR took longer to recover in the SE group. This suggests that the active recovery protocol may have had a positive effect on HRR by potentially improve parasympathetic activation [38]. However, further research is required to show this conclusively.

The mechanisms underpinning the differences between cognitive and physical recovery remain hypothetical. It has been shown that restricting sleep towards the end of the night reduces the proportion of Rapid Eye Movement (REM) while Non-REM (NREM) was conserved [39]. Those adjustments could explained the present results as NREM has been linked to cellular repair and energy storing while REM aims to improve cognitive processes [40]. In this study, the inclusion of an early morning session would have resulted in a reduction of REM proportion and therefore could explain the lack of difference on neuromuscular function as this suggests that the proportion of NREM was similar in both groups. However, results regarding professional football players showed that players went to bed later (i.e. ≈02:00 AM) than the present bedtime (i.e. midnight) observed in this study. While this is a realistic assumption, further research needs to explore the effect of different sleep restriction scenarios (morning vs. evening sleep restriction) and their respective effects on post-exercise recovery.

Nevertheless, it is possible that a more targeted approach to enhance NREM could improve neuromuscular recovery as total sleep time did not seem to affect recovery positively or negatively. This necessity to improve NREM post-exercise is further strengthened by recent findings showing that the contact activity sustained during match may specifically decrease NREM [36]. Further research is required to understand how sleep extension may change sleep architecture and consequently post-recovery kinetics.

### The effects of sleep extension and active recovery at POST 36

While observing residual fatigue 30 hours following contact-based activity is normal [41], the absence of difference in recovery status was surprising. The absence of difference between groups could be due to the similar sleep instructions given to the players on night 2 facilitating CON group to pay back the acute sleep loss sustained. In turn, better subjective sleep quality and total sleep time were observed in CON group on night 2 compared to SE despite similar time in bed. This is characteristic of a sleep rebound observed after sleep restriction suggesting that the sleep pressure was more important in the CON group [42]. Indeed, due to the early scheduling of the recovery session, the CON group slept only 04h30. This behaviour might help CON group to counterbalance the negative effect of sleep restriction and explain the similar recovery status than SE at POST 36. Despite some positive effects at POST 14, active recovery did not show better effect at POST 36 which supports the notion that scheduling early active recovery remains controversial [14]. Practitioners should consequently avoid such practice scheduling and promote regular sleep patterns instead as similar recovery status was observed. This is an important consideration because the cumulative load of these sessions over the course of a season contributes to a substantial burden on staff and player’s time. It is possible that the positive effect observed at POST 14 could have been maintained or accentuated if the sleep extension protocol was prolonged over several nights as shown in previous studies [15, 43, 44]. Nevertheless, the implementation of all of these strategies is carefully related to the general team schedule. Staff members should be aware about the influence that they can have on sleep via the implementation of early recovery session. Future research should consider to extend sleep over the 2 days of the usual recovery cycle or the combination of active recovery and sleep extension in team sport.

### Limitations

Despite the practical usefulness of actigraphy in the sport context, this method did not allow a measure of sleep architecture. Such a method would help to understand the difference between cognitive and physical recovery observed at POST 14 and potential link with sleep stages. Secondly, baseline measurements showed that study participants were not achieving the recommend value of 7-9h of sleep per night [45]. Consequently, it is possible that participants contracted a sleep debt before the study and hide the potential effect of sleep extension. This further suggests that the recommended 10 hours of sleep might needs to be individualized based on the sleep profile (i.e. short vs. long sleeper). Finally, chronotype has not been investigated in this study. Preliminary analysis reported that all participants presented mid-range chronotype and therefore chronotype analysis could not be used in the present investigation. Future studies should consider including different chronotypes (morning vs. evening) and assess how it can impact sleep promoting strategies among athletes and post-exercise recovery.

As it stands, it remains difficult to isolate the exact effect of sleep extension per se on recovery markers. The inclusion of a control group would have helped to ensure the exact effect of sleep extension compared with the active recovery strategy. While the inclusion of a control group remains always problematic in sport science research, the inclusion of active recovery within the sleep extension group would have helped to overcome this limitation. Further research will need to consider the effect of combined recovery (ice bath, active recovery) and sleep strategies on player status recovery.

### Practical applications

- Sleep extension induces positive effects on cognitive function but not on neuromuscular function 14h following training.
- Practitioners should carefully consider the scheduling of recovery session as it could be detrimental to sleep. Therefore, if active recovery is performed, staff members should ensure it will not interfere with sleep.
- At 36h following training, the recovery protocols were equally effective, and therefore the choice of recovery approach is a matter of athlete and practitioner preference.
- Practitioners should be aware that they can have a direct impact on sleep by manipulating player’s training schedule.

## Conclusion

The main results from this study suggest that acute sleep extension strategy or early active recovery impact specific aspects of the recovery process. However due to the acute nature of the intervention, this strategy does not translate to better recovery status over several days. Further research is required to assess how different strategies could impact sleep architecture and post exercise recovery in an elite team sport context.

## Supporting information

S1 Data (ZIP)
